# Metabolomic-genomic prediction can improve prediction accuracy of breeding values for malting quality traits in barley

**DOI:** 10.1186/s12711-023-00835-w

**Published:** 2023-09-05

**Authors:** Xiangyu Guo, Pernille Sarup, Ahmed Jahoor, Just Jensen, Ole F. Christensen

**Affiliations:** 1 Center for Quantitative Genetics and Genomics, Aarhus University, 8000 Aarhus C, Denmark; 2 Danish Pig Research Centre, Danish Agriculture and Food Council, 1609 Copenhagen V, Denmark; 3 Nordic Seed A/S, 8300 Odder, Denmark; 4 Department of Plant Breeding, The Swedish University of Agricultural Sciences, 2353 Alnarp, Sweden

## Abstract

**Background:**

Metabolomics measures an intermediate stage between genotype and phenotype, and may therefore be useful for breeding. Our objectives were to investigate genetic parameters and accuracies of predicted breeding values for malting quality (MQ) traits when integrating both genomic and metabolomic information. In total, 2430 plots of 562 malting spring barley lines from three years and two locations were included. Five MQ traits were measured in wort produced from each plot. Metabolomic features used were 24,018 nuclear magnetic resonance intensities measured on each wort sample. Methods for statistical analyses were genomic best linear unbiased prediction (GBLUP) and metabolomic-genomic best linear unbiased prediction (MGBLUP). Accuracies of predicted breeding values were compared using two cross-validation strategies: leave-one-year-out (LOYO) and leave-one-line-out (LOLO), and the increase in accuracy from the successive inclusion of first, metabolomic data on the lines in the validation population (VP), and second, both metabolomic data and phenotypes on the lines in the VP, was investigated using the linear regression (LR) method.

**Results:**

For all traits, we saw that the metabolome-mediated heritability was substantial. Cross-validation results showed that, in general, prediction accuracies from MGBLUP and GBLUP were similar when phenotypes and metabolomic data were recorded on the same plots. Results from the LR method showed that for all traits, except one, accuracy of MGBLUP increased when including metabolomic data on the lines of the VP, and further increased when including also phenotypes. However, in general the increase in accuracy of MGBLUP when including both metabolomic data and phenotypes on lines of the VP was similar to the increase in accuracy of GBLUP when including phenotypes on the lines of the VP. Therefore, we found that, when metabolomic data were included on the lines of the VP, accuracies substantially increased for lines without phenotypic records, but they did not increase much when phenotypes were already known.

**Conclusions:**

MGBLUP is a useful approach to combine phenotypic, genomic and metabolomic data for predicting breeding values for MQ traits. We believe that our results have significant implications for practical breeding of barley and potentially many other species.

**Supplementary Information:**

The online version contains supplementary material available at 10.1186/s12711-023-00835-w.

## Background

The aim of plant breeding activities is to maximise selection gain per unit of time [1]. To reach this goal, scientific breeding activities aim at ranking early and accurately the selection candidates by their genetic merit, which is estimated by an appropriate experimental design and statistical analysis of their performance [2]. The history of scientific breeding of plants can be traced back to the theoretical basis of Mendel’s inheritance laws and the concepts of evolutionary biology [1, 3]. Following the rediscovery of Mendelian inheritance, many quantitative genetic techniques have become common practice in plant breeding [4]. In practical breeding, measurements of phenotypes are used to inform selection, and generally these measurements must be conducted in well-designed experiments repeated over locations and years to avoid confounding effects from the environment when selection is based on the phenotype alone [2, 5]. However, this traditional phenotypic selection program is time-consuming and expensive, which limits the efficacy of the breeding program. Genomic selection (GS) has shown to be a promising strategy for increasing genetic gain in plant breeding [6]. In GS, dense genome-wide markers and phenotypes from a training population (TP) are used to predict genomic breeding values of candidates based only on genotype data but not phenotype data [7]. A prerequisite for the practical use of GS is the availability of genome-wide markers on many individuals/lines at a low cost [8]. The application of GS makes early selection before phenotypes are collected possible, which can increase the efficiency of the breeding efforts considerably due to shorter generation intervals and larger populations available for selection.

Various statistical models/methods have been proposed in GS, among which a widely used method is genomic best linear unbiased prediction (GBLUP) [9, 10]. Best linear unbiased prediction (BLUP) is a method used in linear mixed models for the prediction of random effects [11, 12], and has been widely applied in animal and plant breeding for ranking individuals/lines in breeding programs, using the correlation between genetic effects that is due to pedigree relationships. In GS, the genetic effects on different individuals/lines are correlated due to the sharing of observed marker alleles, and in GBLUP this is implemented via the construction of a marker-based relationship matrix between individuals/lines that is computed from all available markers.

Similar to dense genome-wide DNA-markers, ‘omics’ data such as transcriptomic and metabolomic data are becoming available in increasingly larger quantities and at decreasing cost. Such ‘omics’ data consist of measurements of effects that are intermediate between the genotype and phenotype of interest for breeding. In recent years, the use of multi-omics data for the prediction of trait phenotypes has been investigated by several studies [13–15]. In these studies, both whole-metabolome effects and whole-transcriptome effects on individuals/lines were assumed to be correlated via similarity matrices, and effects were predicted using BLUP, which in both respects are similar to GBLUP. However, prediction of phenotypes is the prediction of both the genetic and environmental effects, and only the predicted genetic effects are of interest for breeding purposes. Breeding values are the part of genetic effects that are transmitted to the offspring, and these are of primary interest for selecting parental lines. Accurate prediction of breeding values is crucial in plant breeding programs to maximise genetic gain. In order to meet the need for integrating ‘omics’ data into a genetic evaluation system, a joint model for phenotypes and ‘omics’ data was developed by Christensen et al. [16], where methods for the computation of BLUP breeding values from such a model were also described. Martinez Boggio et al. [17] investigated the model and methods on milk production traits and microbiota data as the omics data, but the results showed no increase in accuracy of predicted breeding values when including microbiota data, presumably due to the microbiota data only explaining a small proportion of the variation in the traits of interest. The model and methods in Christensen et al. [16] provide an opportunity for improving accuracies of predicted breeding values in practical plant or animal breeding by incorporating ‘omics’ data for traits that are genetically related to these ‘omics’ data, and especially when the traits of interests are costly to measure or difficult to improve.

Barley (*Hordeum vulgare* L.) is the most common source of malt that is used in brewing alcoholic beverages [18]. Malting quality (MQ) traits are crucial in the practical breeding of malting barley, but the measurement of MQ traits is expensive and labor-intensive [19, 20]. In the whole process of brewing, numerous metabolic processes are involved, which results in distinct and time-dependent alterations in metabolite profiles [21]. Therefore, two previous studies were conducted. The first study investigated the genomic variance in metabolomic profiles extracted from spring barley wort, and the metabolomic features were found to be both heritable and genetically-related to MQ traits, and thus to have a good potential to be used in selection for high MQ [22]. The second study investigated the prediction of phenotypes using metabolomic features and the results showed that these phenotypic predictions were highly correlated to the actual phenotypes in a validation dataset [23]. Keeping these two results in mind, the joint model for phenotypes and ‘omics’ data proposed by Christensen et al. [16] may be an appropriate tool in practical barley breeding for the integration of both genomic and metabolomic information into genetic evaluations.

Therefore, the aim of this study was to investigate the possibility of combining phenotypic, genomic, and metabolomic data for the genetic evaluation of MQ traits in spring barley. To reach this goal, the joint model including both genomic and metabolomic information (MGBLUP) was compared with the baseline genomic model (GBLUP) to estimate variance components (VC) and predict breeding values. The accuracies of predicted breeding values were evaluated with two cross-validation strategies: leave-one-year-out and leave-one-line-out schemes. In addition, the increase in accuracy from the successive inclusion of, first metabolomic data on the validation lines, and second both metabolomic and phenotype data on the validation lines, was assessed using the LR method [24].

## Methods

All data, including phenotypic, genomic, and metabolomic data, used are available in a public accessible repository [25].

### Data

In this study, a dataset on 2430 plots for 562 spring barley malting lines was included. These barley lines and the measurements of MQ traits are part of the current genomic breeding program from Nordic Seed A/S, and represent a subset of the plots analysed in Guo et al. [23], i.e. consisting only of plots with records on all traits and lines with genotype information. We used samples from two locations in Denmark, which were collected from each plot individually, and the data covered three years from 2014 to 2016. At both locations, the fields were divided into trials, which included 52 to 106 smaller plots (8.25 m^2^). Each trial was designed as a randomised complete block comprising 20 to 45 lines with three replicates of each line [26]. Each trial included two control lines with three replicates. As a consequence, testing was conducted in a number of trials within each year-location combination. The malt sample from each plot was milled and extracted in water in order to produce a wort as described in a previous study [20]. The wort was used to measure malting quality (MQ) traits, and here we focused on filtering speed (FS), extract yield (EY), wort color (WC), beta-glucan content (BG), and wort viscosity (WV), which are five of the six traits analysed in Guo et al. [23], excluding one trait that showed little variation (wort clearness, for which 92% of the observations were in the first visually-scored category). A detailed description of these MQ traits is reported in Sarup et al. [20]. Genotypic data were based on the Illumina iSelect9K barley chip and 3889 single-nucleotide polymorphisms (SNPs) were used after editing based on the following criteria: a minor allele frequency higher than 5%, and a proportion of missing markers lower than 20%. The remaining missing genotypes were assigned to the heterozygous genotype. Metabolomic features (MF) were nuclear magnetic resonance (NMR) data expressed as 24,018 intensities obtained from one-dimensional (1D) ^1^H NMR spectra, the intensities were integrated over small chemical shift intervals, expressed in parts per million (ppm) in the frequency range from 0.70 ppm to 9.00 ppm.

### Statistical models and methods

A widely used approach for genetic evaluation is BLUP, which was derived by CR Henderson in 1950 [11, 12] to predict random effects in a statistical linear mixed model. The method has been widely used in animal and plant breeding for ranking the best individuals/lines in breeding programs. In this study, genomic BLUP (GBLUP) and metabolomic-genomic BLUP (MGBLUP) methods were compared.

#### GBLUP

In this study, GBLUP from Guo et al. [22] was applied as a baseline model, specified as follows:

$$\mathbf{y}=\mathbf{X}\mathbf{b}+{\mathbf{Z}}_{g}\mathbf{g}+{\mathbf{Z}}_{g}\mathbf{l}+{{\mathbf{Z}}_{{i}_{g}}\mathbf{i}}_{g}+{{\mathbf{Z}}_{{i}_{l}}\mathbf{i}}_{l}+{\mathbf{Z}}_{t}\mathbf{t}+\mathbf{e}, \qquad (\mathrm{GBLUP})$$

where $\mathbf{y}$ is the vector of records for each MQ trait, $\mathbf{b}$ is the vector of location × year × trial effects to correct for differences caused by experimental location, year, and trial, and all interactions between these effects, $\mathbf{g}$ is the vector of additive genomic effects for each line explained by genomic markers, $\mathbf{l}$ is the vector of line effects for differences between lines that are not explained by additive effects of genomic markers, ${\mathbf{i}}_{g}$ is the vector of additive genotype by environment (six location × year environments) interaction effect which accounts for the additive genetic differences in genotype caused by different location × year environments, ${\mathbf{i}}_{l}$ is the vector of line-by-environmental interaction effect, which accounts for the differences in line caused by different location × year environments but not explained by the additive genotype by environment interaction effect, $\mathbf{t}$ is the vector of effects for batches of samples malted and mashed simultaneously, which accounts for the environmental effects induced by the different batches, $\mathbf{X}$, ${\mathbf{Z}}_{g}$, ${\mathbf{Z}}_{{i}_{g}}$, ${\mathbf{Z}}_{{i}_{l}}$ and ${\mathbf{Z}}_{t}$ are the corresponding incidence matrices for $\mathbf{b}$, $\mathbf{g}$, $\mathbf{l}$, and $\mathbf{t}$, respectively, and $\mathbf{e}$ is the vector of residual effects, i.e. variation that cannot be explained by the other effects in the model. In this model, $\mathbf{b}$ is the vector of fixed effects parameters, and $\mathbf{g}$, $\mathbf{l}$, ${\mathbf{i}}_{g}$, ${\mathbf{i}}_{l}$, $\mathbf{t}$ and $\mathbf{e}$ are the vectors of random effects with $\mathbf{g}\sim N\left(\mathbf{0},\mathbf{G}{\sigma }_{g}^{2}\right)$, $\mathbf{l}\sim N\left(\mathbf{0},\mathbf{I}{\sigma }_{l}^{2}\right)$, ${\mathbf{i}}_{g}\sim N\left(\mathbf{0},diag(\mathbf{G},\cdots \mathbf{G}){\sigma }_{{i}_{g}}^{2}\right)$, ${\mathbf{i}}_{l}\sim N\left(\mathbf{0},\mathbf{I}{\sigma }_{{i}_{l}}^{2}\right)$, $\mathbf{t}\sim N(\mathbf{0},\mathbf{I}{\sigma }_{t}^{2})$, $\mathbf{e}\sim N(\mathbf{0},\mathbf{I}{\sigma }_{e}^{2})$, and these random effects are assumed to be independent of each other. Matrix $\mathbf{G}$ denotes the genomic additive relationship matrix computed using genomic data with VanRaden’s method 1 [9], matrix $diag(\mathbf{G},\cdots \mathbf{G})$ denotes the block-diagonal matrix with $\mathbf{G}$ as block-diagonal elements in the six location × year environments, and $\mathbf{I}$ denotes the identity matrix.

#### MGBLUP

In this study, MGBLUP was used to allow the integration of both metabolomic and genomic data for prediction of breeding values. The model and methods closely follow the development by Christensen et al. [16]. First, we present the model in the context of our study, and second we explain the MGBLUP applied in this study.

##### Metabolomic-genomic model

The model developed by Christensen et al. [16] is a basic model for integrating different ‘omics’ data into genetic evaluations. In our study, we extend this basic model to include additional random effects as in the GBLUP model, and also to incorporate the fact that we have multiple phenotypic and metabolomic records for each genotype. The model that we use is a joint model for phenotypes and metabolomic intensities and is specified by Eqs. (1) and (2) below:

1 $$\mathbf{y}=\mathbf{X}{\mathbf{b}}_{1}+{\mathbf{Z}}_{m}\mathbf{M}{\varvec{\upalpha}}+{\mathbf{Z}}_{g}{\mathbf{g}}_{1}+{\mathbf{Z}}_{g}{\mathbf{l}}_{1}+{\mathbf{Z}}_{{i}_{g}}{{\mathbf{i}}_{g}}_{1}+{\mathbf{Z}}_{{i}_{l}}{{\mathbf{i}}_{l}}_{1}+{\mathbf{Z}}_{t}{\mathbf{t}}_{1}+{\mathbf{e}}_{1},$$

2 $${\mathbf{m}}_{\mathrm{j}}= \mathbf{X}{{\varvec{\upbeta}}}_{j}+{\mathbf{Z}}_{g}{\mathbf{g}}_{j,2}+{\mathbf{Z}}_{g}{\mathbf{l}}_{j,2}+{\mathbf{Z}}_{{i}_{g}}{\mathbf{i}}_{{g}_{j,2}}+{{\mathbf{Z}}_{{i}_{l}}\mathbf{i}}_{{l}_{j,2}}+{\mathbf{Z}}_{t}{\mathbf{t}}_{j,2}+{\mathbf{e}}_{j,2}, \quad j=1,\dots , k$$

where $\mathbf{y}$, $\mathbf{X}$, $\mathbf{b}$, ${\mathbf{Z}}_{g}$, $\mathbf{g}$, ${\varvec{l}}$, ${\mathbf{i}}_{g}$, ${\mathbf{i}}_{l}$, ${\mathbf{Z}}_{t}$, $\mathbf{t}$, $\mathbf{e}$ are defined as for the GBLUP model regardless of the subscripts 1 or 2. Equation (1) describes the relationship between phenotypes and metabolomic intensities. Here, matrix $\mathbf{M}$ contains, as columns, the vectors ${\mathbf{m}}_{1}$ to ${\mathbf{m}}_{k}$ of metabolomic intensities for each of the $k$ features, ${\varvec{\upalpha}}$ is the vector of the regression effects of metabolomic intensities on phenotypes, matrix ${\mathbf{Z}}_{m}$ is an incidence matrix, and ${\mathbf{g}}_{1}$ is the vector of direct genetic effects (termed residual genetic effects in Christensen et al. [16]), which are genetic effects on the phenotypes that are not explained through the observed metabolomic intensities. Equation (2) for metabolomic feature $j=1,\dots ,k$ describes the relationship between metabolomic intensities ${\mathbf{m}}_{j}$ and genetic and environmental effects for the lines. For the $j$ th feature, ${{\varvec{\upbeta}}}_{j}$ is the vector of location × year × trial fixed effects, ${\mathbf{g}}_{j,2}$ is the vector of genetic effects on intensities, ${\mathbf{l}}_{j,2}$ is the vector of line effects on intensities, ${\mathbf{i}}_{{g}_{j,2}}$ is the vector of additive genotype by environment interaction effects on intensities, ${\mathbf{i}}_{{l}_{j,2}}$ is the vector of line by environmental interaction effects on intensities, ${\mathbf{t}}_{j,2}$ is the vector of batch effects on intensities, and ${\mathbf{e}}_{j,2}$ is the vector of residual effects on intensities. All the random effect vectors are independent and their distributions are ${\varvec{\upalpha}}\sim N\left(\mathbf{0},\mathbf{I}{\sigma }_{\alpha }^{2}\right)$, ${\mathbf{g}}_{1}\sim N\left(\mathbf{0},\mathbf{G}{\sigma }_{{g}_{1}}^{2}\right)$, ${\mathbf{l}}_{1}\sim N\left(\mathbf{0},\mathbf{I}{\sigma }_{{l}_{1}}^{2}\right)$, ${\mathbf{i}}_{{g}_{1}}\sim N\left(\mathbf{0},diag(\mathbf{G},\cdots \mathbf{G}){\sigma }_{{i}_{{g}_{1}}}^{2}\right)$, ${\mathbf{i}}_{{\mathrm{l}}_{1}}\sim N\left(\mathbf{0},\mathbf{I}{\sigma }_{{i}_{{l}_{1}}}^{2}\right)$, ${\mathbf{t}}_{1}\sim N(\mathbf{0},\mathbf{I}{\sigma }_{{t}_{1}}^{2})$, ${\mathbf{e}}_{1}\sim N(\mathbf{0},\mathbf{I}{\sigma }_{{e}_{1}}^{2})$, ${\mathbf{g}}_{j,2}\sim N\left(\mathbf{0},\mathbf{G}{\sigma }_{{g}_{j,2}}^{2}\right)$, ${\mathbf{l}}_{j,2}\sim N\left(\mathbf{0},\mathbf{I}{\sigma }_{{l}_{j,2}}^{2}\right)$, ${\mathbf{i}}_{{g}_{j,2}}\sim N\left(\mathbf{0},diag(\mathbf{G},\cdots \mathbf{G}){\sigma }_{{i}_{{g}_{j,2}}}^{2}\right)$, ${\mathbf{i}}_{{l}_{j,2}}\sim N\left(\mathbf{0},\mathbf{I}{\sigma }_{{i}_{j,2}}^{2}\right)$, ${\mathbf{t}}_{j,2}\sim N(\mathbf{0},\mathbf{I}{\sigma }_{{t}_{j,2}}^{2})$, ${\mathbf{e}}_{j,2}\sim N(\mathbf{0},\mathbf{I}{\sigma }_{{e}_{j,2}}^{2})$, $j=1,\dots ,k$. The connection between Eqs. (1) and  (2) is that the model in Eq. (2) is conditional on metabolomic intensities ${\mathbf{m}}_{1}$ to ${\mathbf{m}}_{k}$, and Eq. (2) describes the model for these intensities. As explained in Christensen et al. [16], the vector of breeding values is $\mathbf{a}={\sum }_{j}{\mathbf{g}}_{j,2}{\alpha }_{j}{+\mathbf{g}}_{1}$. In this paper, we denote the first term as the vector of metabolome mediated breeding values, although we are aware that the observed metabolomic data is not the actual metabolome itself. Consequently, the heritability is also the sum of two terms, and we denote the first term as the metabolome-mediated heritability.

##### Prediction of breeding values using MGBLUP

In Christensen et al. [16], it was shown that predicted breeding values in the metabolomic-genomic model can be obtained by solving two mixed model equation systems successively, where each of these systems correspond to a linear mixed model. In our context, this implies that inference in the metabolomic-genomic model can be obtained from successively applying MGBLUP_1_ and MGBLUP_2_ as shown below.

The first step is:

$$\mathbf{y}=\mathbf{X}{\mathbf{b}}_{1}+{\mathbf{Z}}_{m}\mathbf{u}+{\mathbf{Z}}_{g}{\mathbf{g}}_{1}+{\mathbf{Z}}_{g}{\mathbf{l}}_{1}+{{\mathbf{Z}}_{{i}_{g}}{\mathbf{i}}_{g}}_{1}+{\mathbf{Z}}_{{i}_{l}}{{\mathbf{i}}_{l}}_{1}+{\mathbf{Z}}_{t}{\mathbf{t}}_{1}+{\mathbf{e}}_{1}, \qquad (\mathrm{MGBLUP}1)$$

where $\mathbf{u}$ is the vector of metabolomic effects on phenotype,

$$\mathbf{u}=\mathbf{M}{\varvec{\upalpha}},$$

with $\mathbf{M}{\varvec{\upalpha}}$ defined in Eq. (1), and the other effects are the same as in Eq. (1). The metabolomics effects $\mathbf{u}\sim N\left(\mathbf{0},\mathbf{Q}{\sigma }_{u}^{2}\right)$, where $\mathbf{Q}=\frac{\mathbf{M}{\mathbf{M}}^{\mathbf{^{\prime}}}}{q}$, with $\mathbf{M}$ being a $p\times q$ matrix of adjusted, centered and scaled NMR intensities (mean of 0 and standard deviation of 1 as in Guo et al. [23], where further details can be found) with $q$ = 24,018 (equal to the number of MF) and $p$ = 2430 (equal to the number of samples).

The second step is:

$$\widehat{\mathbf{u}}=\mathbf{X}{\mathbf{b}}_{2}+{\mathbf{Z}}_{g}{\mathbf{g}}_{2}+{\mathbf{Z}}_{g}{\mathbf{l}}_{2}+{{{\mathbf{Z}}_{{i}_{g}}\mathbf{i}}_{g}}_{2}+{{{\mathbf{Z}}_{{i}_{l}}\mathbf{i}}_{l}}_{2}+{\mathbf{Z}}_{t}{\mathbf{t}}_{2}+{\mathbf{e}}_{2}, \qquad (\mathrm{MGBLUP}2)$$

where $\widehat{\mathbf{u}}$ is the vector of predicted metabolomics effects from MGBLUP_1_, and the effects are defined similarly to those in Eq. (2). The distribution of random effects are ${\mathbf{g}}_{2}\sim N\left(\mathbf{0},\mathbf{G}{{\sum }_{j}{\sigma }_{{g}_{j,2}}^{2}\sigma}_{\alpha}^{2}\right)$, ${\mathbf{l}}_{2}\sim N\left(\mathbf{0},\mathbf{I}{{\sum }_{j}{\sigma }_{{l}_{j,2}}^{2}\sigma}_{\alpha}^{2}\right)$, ${\mathbf{i}}_{{g}_{2}}\sim N\left(\mathbf{0},{diag(\mathbf{G},\cdots \mathbf{G}){\sum }_{j}{\sigma }_{{i}_{{g}_{j,2}}}^{2}\sigma}_{\alpha}^{2}\right)$, ${\mathbf{i}}_{{l}_{2}}\sim N\left(\mathbf{0},\mathbf{I}{{\sum }_{j}{\sigma }_{{i}_{{l}_{j,2}}}^{2}\sigma}_{\alpha}^{2}\right)$, ${\mathbf{t}}_{2}\sim N\left(\mathbf{0},\mathbf{I}{{\sum }_{j}{\sigma }_{{t}_{j,2}}^{2}\sigma}_{\alpha}^{2}\right)$, ${\mathbf{e}}_{2}\sim N\left(\mathbf{0},\mathbf{I}{{\sum }_{j}{\sigma }_{{e}_{j,2}}^{2}\sigma}_{\alpha}^{2}\right)$, $j=1,\dots ,k$, according to Christensen et al. [16]. For MGBLUP, the predicted breeding values were calculated as the sum of the predicted breeding values from MGBLUP_1_ and MGBLUP_2_, following the derivation in Christensen et al. [16],

3 $$\widehat{\mathbf{a}}=\widehat{{\mathbf{g}}_{1}}+\widehat{{\mathbf{g}}_{2}.}$$

### Estimation of the variance components

The full dataset was used to estimate the variance components (VC) in the models described above. The VC in all three models were estimated by restricted maximum likelihood (REML) using the DMU software package [27]. The relative variance component (RVC), which is the proportion of each VC to the total phenotypic variance (${\sigma }_{P}^{2}$) for each plot, was calculated. The total phenotypic variance was calculated as the sum of the VC in each model.

For GBLUP, ${\sigma }_{P}^{2}=\overline{G}{\sigma }_{g}^{2}+{\sigma }_{l}^{2}+\overline{G}{\sigma }_{{i}_{g}}^{2}+{\sigma }_{{i}_{l}}^{2}+{\sigma }_{t}^{2}+{\sigma }_{e}^{2}$, so that the genomic heritability of GBLUP is ${h}_{GBLUP}^{2}=\overline{G}{\sigma }_{g}^{2}/{\sigma }_{P}^{2}$, where $\overline{G }$ is the average of diagonal elements in the $\mathbf{G}$ matrix.

For MGBLUP, the calculation is ${\sigma }_{{P}_{1}}^{2}=\overline{Q}{\sigma }_{u}^{2}+\overline{G}{\sigma }_{{g}_{1}}^{2}+{\sigma }_{{l}_{1}}^{2}+\overline{G}{\sigma }_{{i}_{{g}_{1}}}^{2}+{\sigma }_{{i}_{{l}_{1}}}^{2}+{\sigma }_{{t}_{1}}^{2}+{\sigma }_{{e}_{1}}^{2}$, where $\overline{Q }$ is the average diagonal of the $\mathbf{Q}$ matrix, thus the direct heritability is ${h}_{d}^{2}=\overline{G}{\sigma }_{{g}_{1}}^{2}/{\sigma }_{{P}_{1}}^{2}$, and the metabolomic variance ratio is ${c}_{m}^{2}=\overline{Q}{\sigma }_{u}^{2}/{\sigma }_{{P}_{1}}^{2}$. Furthermore, according to Christensen et al. [16], the heritability of the metabolomic intensities can be obtained from MGBLUP_2_, i.e. here ${h}_{m}^{2}=\overline{G}{\sigma }_{{g}_{2}}^{2}/(\overline{G}{\sigma }_{{g}_{2}}^{2}+{\sigma }_{{l}_{2}}^{2}+\overline{G}{\sigma }_{{i}_{{g}_{2}}}^{2}+{\sigma }_{{i}_{{l}_{2}}}^{2}+{\sigma }_{{t}_{2}}^{2}+{\sigma }_{{e}_{2}}^{2})$, and therefore according to Christensen et al. [16], the genomic heritability based on the MGBLUP model is: ${h}_{MGBLUP}^{2}={c}_{m}^{2}{h}_{m}^{2}+{h}_{d}^{2}$. For the VC other than the genomic ones, RVC are computed similarly.

It is common in plant breeding [28] to provide a heritability of line mean instead of a heritability of plot records, as is done here. Such estimates for the heritability of line mean will increase with the introduction of an improved experimental design with more years and locations being tested, while the heritability of plot reflects, in a more neutral way, how heritable the traits of interests are, and in addition, in our study the MF were measured for each plot. Therefore, we chose to present the heritability of plot records in this study.

### Cross-validation

Cross-validation was carried out to compare the accuracies of predicted breeding values using GBLUP and MGBLUP. Two different cross-validation schemes, in which the whole population was divided into a training population (TP) and a validation population (VP), were investigated in this study based on two different hypotheses regarding the factors that influence prediction accuracy. The two schemes were leave-one-year-out (LOYO) and leave-one-line-out (LOLO). In LOYO, one out of three years was left out so that the accuracy of predicting one year from the other two years could be investigated. This scheme is similar to the prediction of lines from a new breeding cycle. In LOLO, one out of 562 lines was left out so that the accuracy of predicting one line from all the other lines could be investigated. This scheme is similar to the prediction of lines for which no MQ phenotypes have been measured but the other lines from the same breeding cycle have been phenotyped for MQ.

In each round of the cross-validation, according to the setup for TP size, a certain number of plots were selected, and for the remaining plots both phenotypes and MF were masked (for LOLO, all plots for a given line were masked, and for LOYO, all plots in a given year were masked). The phenotypes of the masked samples were predicted based on the TP together with the genomic information. As a measure of the accuracy of prediction, we report the correlation between phenotypes for each plot corrected for the location × year × trial fixed effect and the random batch effect based on GBLUP and the predicted breeding values from each model, and as an assessment of the dispersion bias we report the regression coefficient of these corrected phenotypes on predicted breeding values for each model. Differences in correlations between GBLUP and MGBLUP were assessed using a Hotelling-Williams t-test [29, 30] at a level of 5%.

The reader should note here that we do not present cross-validation results in the scenario in which only phenotypes in the VP were masked (i.e. metabolomic data in the VP were not masked), although such predicted breeding values are very relevant from a practical view. The reason is that, in this scenario, the correlation between corrected phenotypes and predicted breeding values would be influenced by environmental effects in a similar way to the situation with a bivariate model and records for the secondary trait being available for the lines in the VP (see Additional file 1: Text S1), and therefore this correlation is not an appropriate measure of the accuracy of predicted breeding values.

### Ratios of accuracies and dispersion biases using the LR method

The LR method [24] provides estimators to assess predicted breeding values based on partial (p) and whole (w) datasets. Here, for different scenarios of partial and whole datasets, as described below, we computed ratios of accuracies and dispersion biases based on the vectors of predicted breeding values for the lines in the VP, ${\widehat{\mathbf{a}}}_{p}$ and ${\widehat{\mathbf{a}}}_{w}$, from partial and whole datasets, respectively.

The estimator of the ratio of accuracies is the correlation between ${\widehat{\mathbf{a}}}_{p}$ and ${\widehat{\mathbf{a}}}_{w}$. The expected value of this estimator is the ratio of population accuracies from the partial and whole datasets, and thus, this estimator provides an assessment of the increase in population accuracy when including the additional information in the whole dataset compared to the partial dataset, i.e. a low correlation between ${\widehat{\mathbf{a}}}_{p}$ and ${\widehat{\mathbf{a}}}_{w}$ implies a large increase in accuracy. The estimator of the dispersion is the slope of the regression of ${\widehat{\mathbf{a}}}_{w}$ on ${\widehat{\mathbf{a}}}_{p}$. The expected value of the estimator is 1, indicating no bias in dispersion, whereas a value below 1 indicates over-dispersion and a value above 1 indicates under-dispersion.

**Fig. 1:**
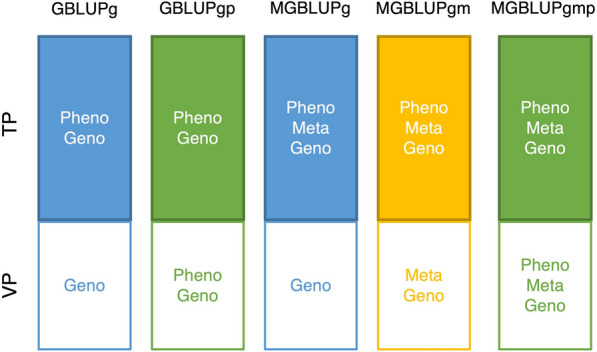
Training and validation population data resources in cross-validation. *GBLUP* genomic best linear unbiased prediction, *MGBLUP*  metabolomics genomic best linear unbiased prediction, *GBLUPg*  GBLUP with genomic data in validation population, *GBLUPgp*  GBLUP with genomic and phenotypic data in validation population, *MGBLUPg*  MGBLUP with genomic data in validation population, *MGBLUPgm*  MGBLUP with genomic and metabolomic data in validation population, *MGBLUPgmp*  MGBLUP with genomic, metabolomic and phenotypic data in validation population, *Pheno*  phenotypic data, *Meta*  metablomic data, *Geno*  genomic data

Partial and whole data are defined according to the TP and VP for the LOYO and LOLO schemes as described in the subsection above on cross-validation. We name the scenarios according to the data resources for the VP as illustrated in Fig. 1, where GBLUPg is GBLUP with genomic and phenotypic data for the TP and genomic data for the VP, GBLUPgp is GBLUP with genomic and phenotypic data for both the TP and VP, MGBLUPg is MGBLUP with genomic, metabolomics and phenotypic data for the TP and genomic data for the VP, MGBLUPgm is MGBLUP with genomic, metabolomics and phenotypic data for the TP and both genomic and metabolomics data for the VP, and MGBLUPgmp is MGBLUP with genomic, metabolomic and phenotypic data for both the TP and VP.

## Results

In this study, first the VC and heritabilities of MQ traits were estimated. Then, the breeding values of MQ traits were predicted by using GBLUP and MGBLUP, and these were assessed using cross-validation, and ratios of population accuracies of predicted breeding values were computed using the LR method.

### Descriptive statistics for malting quality traits

Table 1 shows the descriptive statistics for all the MQ traits analysed in this study. In total, 2430 records were analysed for five MQ traits: filtering speed (FS), extract yield (EY), wort color (WC), beta-glucan content (BG), and wort viscosity (WV). The averages were 4.84 for FS, 82.61 for EY, 5.87 for WC, 217.98 for BG, and 1.47 for WV. The coefficient of phenotypic variation ranged from 2.23% for EY to 52.91% for BG.

**Table 1:** Descriptive statistics for malting quality traits

Trait	N-obs	Unit	Average	Variance	Min	Max	CV
FS	2430	cm/20 min	4.84	0.38	2.30	6.30	12.81%
EY	2430	%	82.61	3.39	70.38	92.39	2.23%
WC	2430	EBC units	5.87	0.71	3.59	8.99	14.31%
BG	2430	mg/L	218	13,303	70	752	52.91%
WV	2430	mPa·s	1.47	0.0036	1.29	1.73	4.08%

*FS*  filtering speed, *EY*  extract yield, *WC*  wort color, *BG*  beta-glucan, *WV*  wort viscosity, *N-obs*  number of observations, *CV* coefficient of phenotypic variation, *EBC* European Brewery Convention

### Estimated variance components

Estimates of genomic and metabolomic variances for GBLUP and MGBLUP are in Table 2. First, we see that the estimated direct genomic variance from MGBLUP is substantially lower than the estimated genomic variance from GBLUP. This is most pronounced for BG, for which this variance decreased by 87% from 2630 to 348, but also for FS, WC and WV, for which the estimated direct genomic variance from MGBLUP decreased by more than 50% compared to the estimated genomic variance from GBLUP (0.014 to 0.005 for FS, 0.14 to 0.04 for WC and 0.0004 to 0.0001 for VW), and only the variance for EY showed a small decrease (0.29 to 0.22). Second, the estimated metabolomic variance from MGBLUP is very large. For all five traits, the estimated metabolomic variance is more than 50% of the total variance from MGBLUP, i.e. 52% for EY, 67% for FS, 91% for WV, 95% for WC and 96% for BG, and for WC, BG and WV, it was even substantially larger than the variance of the phenotype in Table 1 (about four times larger for WC and VW and eight times larger for BG). Third, for all the traits, the total phenotypic variance from GBLUP is much smaller than both the total phenotypic variance from MGBLUP and the variance of phenotype in Table 1, and for all traits except EY, the total phenotypic variance from MGBLUP is much larger than the variance of phenotype in Table 1. Estimates of all other VC for GBLUP and MGBLUP_1_ are shown in Additional file 2: Table S1.

**Table 2:** Estimated genomic and metabolomic variances for malting quality traits using GBLUP and MGBLUP

Trait	GBLUP		MGBLUP				
	$\overline{G}{\sigma }_{g}^{2}$( ${h}^{2}$)	Total	$\overline{G} {\sigma }_{g,1}^{2}$ (${h}_{d}^{2}$)	$\overline{Q}{\sigma }_{u}^{2}$(${c}_{m}^{2}$)	Total	${h}_{m}^{2}$	${h}^{2}$
FS	0.014 (0.06)	0.236	0.005 (0.01)	0.345 (0.67)	0.516	0.17	0.12
EY	0.29 (0.17)	1.68	0.22 (0.07)	1.65 (0.52)	3.17	0.23	0.19
WC	0.14 (0.28)	0.50	0.04 (0.01)	3.15 (0.95)	3.32	0.28	0.28
BG	2625 (0.23)	11,636	348 (0.00)	111,868 (0.96)	116,102	0.27	0.27
WV	0.0004 (0.15)	0.0025	0.0001 (0.01)	0.0156 (0.91)	0.0170	0.25	0.24

*GBLUP*  genomic best linear unbiased prediction, *MGBLUP*  metabolomic-genomic best linear unbiased prediction, *FS*  filtering speed, *EY*  extract yield, *WC*  wort color, *BG*  beta-glucan, *WV*  wort viscosity

$\overline{G}{\sigma }_{g}^{2}$: estimated genomic variance for GBLUP (${h}^{2}$ is the heritability estimate for GBLUP, i.e. proportion of total phenotypic variance); total: total phenotypic variance (GBLUP); $\overline{G} {\sigma }_{g,1}^{2}$: estimated direct genomic variance for MGBLUP (${h}_{d}^{2}$ is the estimated direct heritability for MGBLUP, i.e. proportion of total phenotypic variance); $\overline{Q}{\sigma }_{u}^{2}$: estimated metabolomic variance for MGBLUP (${c}_{m}^{2}$ is the estimated metabolomic variance ratio for MGBLUP, i.e. proportion of total phenotypic variance); total: total phenotypic variance (MGBLUP); ${h}_{m}^{2}$: estimated heritability of metabolomic intensities; ${h}^{2}$: estimated heritability for MGBLUP, ${h}^{2}={c}_{m}^{2}{h}_{m}^{2}+{h}_{d}^{2}$. Standard errors on estimates are in Additional file 2: Tables S1 and S2)

Estimated heritabilities are in Table 2. The estimated genomic heritabilities (${\widehat{h}}^{2}$), which are also RVC for genomic effects, ranged from 0.06 (FS) to 0.28 (WC) when using GBLUP. When metabolomic data were included via the MGBLUP model, the ${\widehat{h}}^{2}$ increased for all five MQ traits, and ranged from 0.12 (FS) to 0.28 (WC). The ${\widehat{h}}^{2}$ estimated from MGBLUP is the sum of a metabolome-mediated part and a direct part, and the decomposition of ${\widehat{h}}^{2}$ based on MGBLUP is in Table 2. As shown in Table 2, the direct ${\widehat{h}}_{d}^{2}$ ranged from 0.00 (BG) to 0.07 (EY), thus the metabolome-mediated heritability accounted for most of the ${\widehat{h}}^{2}$. Estimates of RVC from GBLUP and MGBLUP for all model components are shown in Additional file 3: Fig. S1] and Additional file 2: Table S2.

### Predicted breeding values

LOLO and LOYO cross-validation schemes were investigated for GBLUP and MGBLUP. For the lines in the VP, correlations between predicted breeding values and corrected phenotypes are in Tables 3 and 4 for the LOLO and LOYO schemes, respectively. The general picture for both schemes is that the results from MGBLUP and GBLUP are similar, except for BG and LOYO for which MGBLUP (cor = 0.22) is more accurate than GBLUP (cor = 0.19), and for WV and LOYO for which MGBLUP (cor = 0.12) is slightly more accurate than GBLUP (cor = 0.11). Comparison of the results with the LOLO and LOYO schemes shows that the correlations to corrected phenotypes are much larger for the LOLO than for the LOYO scheme. The regression coefficients for the LOLO and LOYO schemes are also shown in Tables 3 and 4, respectively. These coefficients are based on a regression of corrected phenotypes on predicted breeding values, and the expectation is that they should equal 1. For the LOLO scheme, the coefficients are similar between MGBLUP and GBLUP, and in general reasonably close to 1. For the LOYO scheme and the FS and EY traits, the regression coefficients are all close to 1 for both GBLUP and MGBLUP. However, for the LOYO scheme and the WC, BG and WV traits, the regression coefficients deviate substantially from 1, in particular for WV with a coefficient of 0.42 for GBLUP and 0.56 for MGBLUP.

**Table 3:** Predictive performance of GBLUP and MGBLUP for the LOLO scheme

Trait	GBLUPg		MGBLUPg	
	cor	reg	cor	reg
FS	0.18	1.00	0.18	1.04
EY	0.32	1.02	0.32	1.07
WC	0.46	1.05	0.46	1.05
BG	0.38	1.09	0.38	1.15
WV	0.34	1.13	0.34	1.17

*GBLUP*  genomic best linear unbiased prediction; *MGBLUP*  metabolomic-genomic best linear unbiased prediction; *LOLO*  leave one line out

*FS*  filtering speed, *EY*  extract yield, *WC*  wort color, *BG*  beta-glucan, *WV*  wort viscosity

GBLUPg is GBLUP incorporating genotypes on lines in VP, and MGBLUPg is similarly defined

“cor” columns show the correlations between predicted breeding values and corrected phenotypes for lines in VP, and “reg” columns show the regression coefficients of corrected phenotypes on predicted breeding values. For all traits, none of the differences between the two correlations are statistically significant using a Hotelling-Williams t-test at a 5% level. Standard error on regression coefficient is 0.01 in all cases

**Table 4:** Predictive performance of GBLUP and MGBLUP for the LOYO scheme

Trait	GBLUPg		MGBLUPg	
	cor	reg	cor	reg
FS	0.13	0.95	0.13	1.19
EY	0.27	0.98	0.27	1.05
WC	0.29	0.75	0.30	0.75
BG	0.19*	0.63	0.22^*^	0.86
WV	0.11*	0.42	0.12*	0.56

*GBLUP*  genomic best linear unbiased prediction; *MGBLUP*  metabolomic-genomic best linear unbiased prediction; *LOYO*  leave one year out

*FS*  filtering speed, *EY*  extract yield, *WC*  wort color, *BG*  beta-glucan, *WV*  wort viscosity

GBLUPg is GBLUP incorporating genotypes on lines in VP, and MGBLUPg is similarly defined

“cor” columns show the correlations between predicted breeding values and corrected phenotypes for lines in VP, and “reg” columns show the regression coefficients of corrected phenotypes on predicted breeding values

*Denotes that for the given trait, the difference between the two correlations is statistically significant using a Hotelling-Williams t-test at a 5% level. Standard error on regression coefficient is 0.02 in all cases

The ratios of the population accuracies of predicted breeding values obtained with the LR method with the partial and whole datasets, respectively, are in Tables 5 and 6 for the LOLO and LOYO schemes, respectively. We see that the ratios of the accuracies between GBLUPg and GBLUPgp and between MGBLUPg and MGBLUPgmp are similar for all traits in both the LOLO (ratios ranging from 0.91 for WV to 0.93 for EY) and LOYO (ratios ranging from 0.60 for WV to 0.79 for EY) schemes. We also see that the ratios for the LOYO scheme are smaller than those for the LOLO scheme for all traits and methods. For MGBLUP, the ratios between MGBLUPg and MGBLUPgm are smaller or similar (ranging from 0.97 to 0.98 for LOLO, and from 0.81 to 0.88 for LOYO) than the ratios between MGBLUPgm and MGBLUPgmp (ranging from 0.95 to 0.97 for LOLO, and from 0.80 to 0.89 for LOYO), except for EY, for which the opposite is observed (the first ratio is 1.00 for LOLO and 0.99 for LOYO, and the second ratio is 0.94 for LOLO and 0.81 for LOYO). For all traits, the product of the ratios between MGBLUPg and MGBLUPgm and of the ratio between MGBLUPgm and MGBLUPgmp is slightly smaller than the ratio between MGBLUPg and MGBLUPgmp, i.e. the estimates of these three ratios are not completely consistent with each other because they are estimated independently. The regression coefficients obtained from the LR method (see Additional file 3: Tables S3 and S4] are closer to 1 than those based on cross-validation, but generally show the same patterns.

**Table 5:** Ratios of population accuracies of predicted breeding values for malting quality traits using GBLUP and MGBLUP (LOLO scheme)

Trait	GBLUP-g/gp	MGBLUP-g/gm	MGBLUP-gm/gmp	MGBLUP-g/gmp
FS	0.93	0.95	0.97	0.92
EY	0.93	1.00	0.94	0.93
WC	0.93	0.97	0.97	0.93
BG	0.93	0.95	0.98	0.92
WV	0.91	0.95	0.97	0.91

*GBLUP*  genomic best linear unbiased prediction; *MGBLUP*  metabolomic-genomic best linear unbiased prediction; *LOLO*  leave one line out

*FS*  filtering speed, *EY*  extract yield, *WC*  wort color, *BG*  beta-glucan, *WV*  wort viscosity

GBLUP-g/gp is the ratio of population accuracies for lines in VP from GBLUPg and GBLUPgp, where GBLUPg is GBLUP incorporating genotypes on lines in VP, and GBLUPgp is GBLUP incorporating genotypes and phenotypes on lines in VP. MGBLUP-g/gm, MGBLUP-gm/gmp and MGBLUP-g/gmp are similarly defined with “gm” denoting genotypes and metabolomics on focal lines, and “gmp” denoting genotypes, metabolomics and phenotypes on lines in VP

**Table 6:** Ratios of accuracies of predicted breeding values for malting quality traits using GBLUP and MGBLUP (LOYO scheme)

Trait	GBLUP-g/gp	MGBLUP-g/gm	MGBLUP-gm/gmp	MGBLUP-g/gmp
FS	0.73	0.84	0.87	0.73
EY	0.78	0.99	0.81	0.79
WC	0.71	0.88	0.89	0.71
BG	0.69	0.81	0.88	0.71
WV	0.59	0.81	0.80	0.60

*GBLUP*  genomic best linear unbiased prediction; *MGBLUP*  metabolomic-genomic best linear unbiased prediction; *LOYO*  leave one year out

*FS*  filtering speed, *EY*  extract yield, *WC*  wort color, *BG*  beta-glucan, *WV*  wort viscosity

GBLUP-g/gp is the ratio of population accuracies for lines in VP from GBLUPg and GBLUPgp, where GBLUPg is GBLUP incorporating genotypes on lines in VP, and GBLUPgp is GBLUP incorporating genotypes and phenotypes on lines in VP. MGBLUP-g/gm, MGBLUP-gm/gmp and MGBLUP-g/gmp are similarly defined with “gm” denoting genotypes and metabolomics on focal lines, and “gmp” denoting genotypes, metabolomics and phenotypes on lines in VP

## Discussion

For all the traits, we see that the metabolome-mediated heritability is substantial. Cross-validation results show that, in general, the accuracies of MGBLUP and GBLUP are similar when phenotypes and metabolomic data are recorded on the same plots, and for all traits, except one, and the results of the LR method show that the accuracy of MGBLUP increases when including MF in the VP. Comparing the estimates of the genetic parameters in GBLUP and MGBLUP, we see that, for all the traits, the direct genomic variance from MGBLUP is substantially smaller than the genomic variance from GBLUP, this being most pronounced for BG, but also for FS, WC and WV, for which it is decreased by more than 50%. This pattern is also reflected by the large proportion of estimated metabolomic variance for all traits, being largest for BG (96%), but also very large for FS, WC and WV. Consequently, very large proportions of the heritabilities are mediated by the metabolome. These large proportions of metabolome-mediated heritability are also consistent with one of our previous studies, which found significant genetic correlations between MF and MQ traits [22]. Taken together this implies that the part of breeding values mediated by the metabolome is substantial for these traits, and is largest for BG and smallest for EY.

For MGBLUP, both the metabolomic variance and the total phenotypic variance are large for all the traits. Such large metabolomic variances were also reported by Guo et al. [22]. For all the traits, the total phenotypic variance is larger for MGBLUP than for GBLUP, which is reasonable, since the MF may capture environmental effects that would otherwise be captured by the location × year × trial fixed effect. Usually, the total phenotypic variance from a model is smaller than the variance of the phenotype, since part of the variation would be captured by the fixed effects in the model, and this is also seen in our results from GBLUP for all the traits. Thus, it is surprising that for all the traits, except for EY, the total phenotypic variance from MGBLUP is larger than the variance of the phenotype. A better understanding of the relationship between the total phenotypic variances from GBLUP and MGBLUP_1_, and the variance of the phenotype is needed. In Additional file 4: Text S2, formulas are derived for the first two moments of the variance of the phenotype, ${S}_{y}^{2}$, i.e. expectation and variance of ${S}_{y}^{2}$, in the situation with no fixed effects. First, the expectation of ${S}_{y}^{2}$ for a given model is equal to the total phenotypic variance from the same model. Second, the variance of ${S}_{y}^{2}$ from MGBLUP_1_, with matrix $\mathbf{Q}$ and its associated estimated variance component from our results, is much larger than the variance of ${S}_{y}^{2}$ from GBLUP with matrix $\mathbf{G}$ and its associated estimated variance component from our results. Therefore, a large difference between total phenotypic variance and ${S}_{y}^{2}$ is unrealistic, and taking into account that the phenotypic variance is reduced when including fixed effects, as in the models used in this paper, then the total phenotypic variance being much larger than ${S}_{y}^{2}$ is unrealistic. However, the variance of ${S}_{y}^{2}$ needs to be taken into account, and since that from MGBLUP_1_ is very large, we consider that these very large total phenotypic variances from MGBLUP are not entirely implausible. However, we still do not have a complete understanding of this phenomenon.

Generally, a large total phenotypic variance from MGBLUP could also be the sign of possible deficiencies of the model. One deficiency is the assumption of independence of the MF as postulated by Eq. (2), which is violated since a specific metabolite would often correspond to several MF, and that MF model mediation pathways that are unlikely to be completely independent. In principle, the model in Eqs. (1) and (2) could be extended to incorporate both the genetic covariances between the different MF, and the covariances between the direct genetic effect, ${\mathbf{g}}_{1}$, and the effects explained through the observed metabolomic intensities, ${\mathbf{g}}_{j,2}$, $j=1,\dots ,k$ (implying that the procedure for predicting breeding values, MGBLUP_1_ and MGBLUP_2_, no longer applies, and also that more parameters have to be estimated). However, this deficiency refers to the joint model for the phenotype and MF in Eqs. (1) and (2), whereas the large total phenotypic variances reported in this paper were computed from MGBLUP_1_, i.e. from the model for phenotypes conditional on MF in Eq. (1). Another possible deficiency of Eq. (1) is the assumption of additivity of metabolomics effects, which implies that matrix $\mathbf{Q}$ has a quadratic form. Models with alternative forms of matrix $\mathbf{Q}$, and more generally, the possible deficiencies of the models used here, seem worth investigating further in relation to the total phenotypic variance.

The heritabilities of MF in this study are assumed to be constant across features, i.e. one common heritability, and this is an assumption that is needed when using the two models MGBLUP_1_ and MGBLUP_2_ for the prediction of breeding values [16]. Here, we estimated this common heritability using REML in MGBLUP_2_, as suggested in Christensen et al. [16], and the results show estimates of heritability ranging from 0.17 to 0.28 depending on the trait. According to Eq. (2), this estimated heritability should not actually depend on the trait, and this approach for its estimation is an approximation. In addition, an estimate ranging from 0.17 to 0.28 may seem large compared to the results of Guo et al. [22], who estimated heritabilities for individual MF ranging from 0 to 0.38 with an average of 0.025, and found only 0.3% of the MF having an estimate larger than 0.2. However, such a trait-dependent estimate of the common heritability of MF is actually an estimate of the heritability of a weighted sum of MF, where the weights on MF are the estimated regression effects of metabolomic intensities on phenotypes in Eq. (1). Consequently, MF that do not influence the actual trait are down-weighted, and this seems to be a desirable property. A better understanding of the implications of assuming a common heritability for MF and the development of efficient approaches to estimate this parameter would be useful.

The model in Eqs. (1) and (2) can easily be modified to allow different heritabilities for different MF. The approach used for prediction in MGBLUP_1_ and MGBLUP_2_ could be extended by splitting the metabolomic effects into two components with two different heritabilities, i.e. two similarity matrices constructed from MF that have a high and low heritability, respectively, similar to the extensions of GBLUP with two genomic components [16]. This would require a preliminary step in the analysis, with a screening of MF to decide which have a low or a high heritability. Alternatively, a full Bayesian approach where heritabilities are assigned a prior distribution would be a possibility, for example using the framework in Zhao et al. [31]. Models that allow different heritabilities for MF would be an interesting topic for future research.

Predictive performances of GBLUP and MGBLUP were investigated by cross-validation using the LOYO and LOLO schemes. In the LOLO scheme, the aim was to predict breeding values of a barley line using the phenotypes of all the other lines, and hence the size of the TP was larger than in the LOYO scheme. In addition, lines are generally much more related within year than across years, which causes a stronger genetic relationship between TP and VP in the LOLO than in the LOYO scheme. Therefore, it was expected that a higher prediction accuracy could be obtained from LOLO than from LOYO, and the results obtained are consistent with this expectation. The results also show that prediction accuracies from the LOLO scheme for GBLUP and MGBLUP are similar, with nearly no dispersion bias for either GBLUP or MGBLUP. Hence, incorporating MF for the phenotyped plots, does not increase the accuracy of lines without phenotypes and MF. By combining these results with the results from the LR method, which shows that the ratios for MGBLUPgmp and MGBLUPg are the same as those for GBLUPgmp and MGBLUPg, we also see that incorporating MF for the phenotyped plots does not increase the accuracy of lines with phenotypes. The predictions from the LOLO scheme have less practical importance than those from the LOYO scheme, because, in practical breeding, we usually want to predict one year ahead.

The LOYO scheme mimics a practical breeding system, since a new set of lines should be developed every year. Here, the prediction accuracies of GBLUP and MGBLUP are similar, except for BG and WV, which have higher prediction accuracies in MGBLUP than GBLUP, with the increase in accuracy being largest for BG. These results are consistent with BG being the trait with the largest proportion of estimated metabolomic variance, and also with the proportion of estimated metabolomic variance being large for WV. Regarding dispersion biases, there were only small differences between GBLUP and MGBLUP, but we observed very strong dispersion biases, in particular, for WV, but also for BG (primarily with GBLUP) and WC. Apparently, prediction across years does not work well for these traits and this dataset. An explanation may be that with only two years in the TP, the representation of the variation between performances of lines under different environmental conditions is insufficient for these two years. This interpretation is supported by the results in Table 3 in Sarup et al. [20], who found less dispersion bias for these traits with GBLUP and LOYO in a dataset consisting of one additional year, one additional location, and additional records in 2015 for the two locations in our study. Similar to the LOLO results, the combination of the results of prediction accuracy from the LOYO scheme with the results from the LR method of the ratios for MGBLUPgmp and MGBLUPg that are the same as the ratios for GBLUPgmp and MGBLUPg for LOYO shows that we do not gain much in accuracy by incorporating MF on the same plots for which we do have recorded phenotypes (whether they are own phenotypes and metabolomic data on lines or not).

For MGBLUP and LOYO, a very important question for practical breeding is to assess the accuracy of predicted breeding values of the lines without phenotypic records (either because they are to be obtained later, or are too expensive to record on many lines) based on metabolomic samples (i.e. metabolomic data on the VP, but no phenotypic records on the VP), but as explained in the Methods section this cannot be assessed using cross-validation. An increase in accuracy from the inclusion of metabolomic data on lines in the VP can be assessed from the results of the ratios of accuracies by the LR method as follows. First, the accuracy from MGBLUPg can be computed by dividing the correlation between the predicted breeding values and corrected phenotypes in Table 4 by the square root of the genomic heritability of corrected phenotypes (calculated from the estimated variances from GBLUP in Additional file 2: Table S1); Second, the increase in accuracy of predicted breeding values when including metabolomic data can be assessed by multiplying one minus the ratio of accuracies from MGBLUPg and MGBLUPgm by the accuracy from MGBLUPg. Computing these numbers, we obtain an increase in accuracy when including metabolomic data on lines in the VP from 0.46 to 0.54 for FS, from 0.53 to 0.59 for WC, from 0.44 to 0.53 for BG and from 0.25 to 0.29 for WV and no increase in accuracy 0.62 for EY. The increase in accuracy due to the inclusion of both metabolomic data and phenotypes on lines in the VP can be computed similarly from the ratio of accuracies from MGBLUPg and MGBLUPgmp. When including both metabolomic data and phenotypes on lines in the VP, we obtain an increase in accuracy from 0.46 to 0.59 for FS, from 0.62 to 0.75 for EY, from 0.53 to 0.68 for WC, from 0.44 to 0.57 for BG and from 0.25 to 0.35 for WV. Note that for all the traits, a slightly smaller increase in accuracy would have been obtained when including both metabolomic data and phenotypes, if instead of the ratio between MGBLUPg and MGBLUPgmp, we had used the ratio between MGBLUPgm and MGBLUPgmp, since the estimates of these three ratios of accuracies are not completely consistent with each other. We conclude that for all the traits except EY, there is an increase in accuracy of the lines in the VP when metabolomic data on the lines in the VP are included, and this increase is roughly similar to the subsequent increase when both metabolomic data and phenotypes are included. In other words, except for EY, a substantial proportion of the increase in accuracy that could be obtained from having own records on plots, can be obtained from incorporating metabolomic data on these plots instead, i.e. phenotypic records of MQ can be replaced by metabolomic data for a proportion of the plots.

If we focus on the differences between the results for the MQ traits, we see that EY was the trait with the lowest proportion of variance explained by MF, and also the trait for which inclusion of metabolomics data on the lines in the VP without recorded phenotypes did not increase the accuracy of its predicted breeding value; and BG was the trait with the largest proportion of variance explained by metabolomics, and also the trait for which the inclusion of metabolomic data on the plots with phenotypes increased the accuracy of its predicted breeding value. The actual reasons for these differences in patterns between phenotypes are not known to us. However, we note that EY is a measure of the total concentration of fermentable and non-fermentable sugars, and since the dominant metabolites in the NMR spectra are sugars, then the normalisation of the NMR spectra could result in the removal of important differences related to EY. We also note that the BG trait is actually a metabolite in itself, which is represented in the NMR spectra in several MF.

If we focus on the practical relevance of these results, we note that NMR was recorded on malt, and therefore only available after the plots were harvested and the samples malted. Using these MF for the prediction of lines for which the MQ traits are not recorded could be economically beneficial since the cost to record MQ traits could be avoided, but they do not represent a situation where the selection decision can be done in the early generations of a breeding cycle to decrease generation time and increase selection intensity. To do this, we need to be able to carry out non-destructive sampling, e.g. by sampling leaf cuts. Investigating the performance of MGBLUP or similar models in a situation where MF were observed on growing plants would be very relevant.

In recent years, the integration of multi-omics data has received much interest for predicting the phenotypes of traits [13–15]. In our previous study, we found that the prediction accuracy for MQ phenotypes using MF was very high [23]. However, the core of animal and plant breeding programs is the prediction of breeding values, and our study is about an implementation of a joint model for prediction of breeding values by integrating ‘omics’ data into genetic evaluation. The successful implementation of such a joint model is very relevant to practical plant breeding where traits can be improved by selecting lines with high genetic potential. The MGBLUP model that was applied in the current study would also be applicable for integrating other ‘omics’ data such as transcriptomics, and therefore it also could be interesting to expand the data resources integrated in the genetic evaluation of malting barley.

## Conclusions

In conclusion, we have demonstrated that MGBLUP is a useful approach for combining phenotypic, genomic and metabolomic data for the prediction of the breeding values of MQ traits in barley. We believe that the results from the current study have significant implications for practical breeding of barley and potentially many other species.

### Supplementary Information

**Additional file 1: Text S1.** Predictive correlation with records on a secondary trait in the validation population. Derivations show that in this situation, predictive correlation is not an appropriate measure of the accuracy of predicted breeding values.

**Additional file 2: Table S1.** Variance components from GBLUP and MBLUP_1_ for malting quality traits. **Table S2.** Relative variance components from GBLUP, MGBLUP_1_ and MGBLUP for malting quality traits. **Table S3.** Regression coefficients of predicted breeding values from whole data on predicted breeding values from partial data, for malting quality traits using GBLUP and MGBLUP (LOLO scheme). **Table S4.** Regression coefficients of predicted breeding values from whole data on predicted breeding values from partial data, for malting quality traits using GBLUP and MGBLUP (LOYO scheme).

**Additional file 3: Figure S1.** Proportion of total phenotypic variance explained by each component in malting quality traits.

**Additional file 4: Text S2.** Deviation between empirical variance and total phenotypic variance. Derivations show that the variance of empirical variance is much larger for MGBLUP than for GBLUP.

## Data Availability

All data including metabolomic, genomic as well as phenotypic data are available in a public accessible repository Mendeley Data (https://doi.org/10.17632/s3s4ft92wj.1) which can be accessed through "https://data.mendeley.com/datasets/s3s4ft92wj".
