# Supplementary material for: Metabolomic-genomic prediction can improve prediction accuracy of breeding values for malting quality traits in barley
Source: Genet Sel Evol. 2023 Sep 5;55:61. doi: 10.1186/s12711-023-00835-w (PMC10478459; doi:10.1186/s12711-023-00835-w)
Supplement: Supplementary file 1 — Additional file 1: Text S1. Predictive correlation with records on a secondary trait in the validation population. Derivations show that in this situation, predictive correlation is not an appropriate measure of the accuracy of predicted breeding values. [file 12711_2023_835_MOESM1_ESM.docx]

**Additional file 1**

**Metabolomic-Genomic prediction can improve prediction accuracy of breeding values for malting quality traits in barley**

Xiangyu Guo^1, 2^, ﻿Pernille Sarup^3^, Ahmed Jahoor^3, 4^, Just Jensen^1^, Ole Fredslund Christensen^1*^

^1^ Center for Quantitative Genetics and Genomics, Aarhus University, 8000 Aarhus C, Denmark

^2^ Danish Pig Research Centre, Danish Agriculture & Food Council, 1609 Copenhagen V, Denmark

^3^ Nordic Seed A/S, 8300 Odder, Denmark

^4^ Department of Plant Breeding, The Swedish University of Agricultural Sciences, 2353 Alnarp, Sweden

^*^ Corresponding author:

**Predictive correlation with records on a secondary trait in the validation population.**

When the empirical correlation $\rho(\widehat{\mathbf{a}}^{1},\mathbf{y}^{1})$ is used for evaluating the accuracy of predicted breeding values from different models, we implicitly assume that the predictive correlation is approximately proportional to the accuracy of predicted breeding values,

$$\rho(\widehat{\mathbf{a}}^{1},\mathbf{y}^{1})\approx c\rho(\widehat{\mathbf{a}}^{1},\mathbf{a}^{1}),$$

where $\rho(\widehat{\mathbf{a}}^{1},\mathbf{a}^{1})$ is the empirical correlation between predicted and true breeding values, i.e. the accuracy of predicted breeding values, and the proportionality constant $c$ is the same for all models. We now demonstrate that this property does not hold for situation with records on the secondary trait available for validation individuals.

For the bivariate model, we study a simple model with constant means, genetic effects and residual effects on the two traits, and assuming unrelated individuals. In other words, the model for the primary trait (trait 1) and secondary trait (trait 2) is

$$y_{i}^{1}=\mu^{1}+a_{i}^{1}+e_{i}^{1},$$

$$y_{i}^{2}=\mu^{2}+a_{i}^{2}+e_{i}^{2},$$

with genetic variances $\sigma_{a,11}$ and $\sigma_{a,22}$, residual variances $\sigma_{e,11}$ and $\sigma_{e,22}$, genetic covariance $\sigma_{a,12}$, and residual covariance $\sigma_{e,12}$. Genetic effects $a_{i}^{1},a_{i}^{2}$ are assumed to be independent of residual effects $e_{i}^{1},e_{i}^{2}$ effects for different individuals are assumed to be independent, and all effects are assumed to follow normal distributions.

We assume that all parameter values are known, only records on the secondary trait are available, and breeding values of the primary trait are of interest. It is well known (Mrode, 2015, section 1.6) that the predicted breeding value for individual $i$ is

$$\hat{a}_{i}^{1}=b(y_{i}^{2}-\mu_{2}),$$

where $b=\sigma_{a,12}/(\sigma_{a,22}+\sigma_{e,22})$. In other words, predicted breeding value is co-heritability times trait deviation of the secondary trait.

For validation, we would use records on the primary trait. The empirical correlation between phenotypes and predicted breeding values equals

$$\rho(\widehat{\mathbf{a}}^{1},\mathbf{y}^{1})=bSSD_{12}/\sqrt{SSD_{1}b^{2}SSD_{2}}=SSD_{12}/\sqrt{SSD_{1}SSD_{2}},$$

where $SSD_{12}=\sum_{i} (y_{i}^{1}-\overline{y^{1}})(y_{i}^{2}-\overline{y^{2}})$, $SSD_{12}=\sum_{i} (y_{i}^{1}-\overline{y^{1}})(y_{i}^{2}-\overline{y^{2}})$, $SSD_{12}=\sum_{i} (y_{i}^{1}-\overline{y^{1}})(y_{i}^{2}-\overline{y^{2}})$, with $\overline{y^{1}}=\sum_{i} y_{i}^{1}/n$, $\overline{y^{2}}=\sum_{i} y_{i}^{2}/n$, and $n$ being the number of records.

The accuracy

$$\rho(\widehat{\mathbf{a}}^{1},\mathbf{a}^{1})=bSSD_{y^{2},a^{1}}/\sqrt{SSD_{a^{1}}b^{2}SSD_{2}}=SSD_{y^{2}a^{1}}/\sqrt{SSD_{a^{1}}SSD_{2}}.$$

Now $SSD_{1}$ and $SSD_{a^{1}}$ do not depend on the model, and we also see that for both $\rho(\widehat{\mathbf{a}}^{1},\mathbf{y}^{1})$ and $\rho(\widehat{\mathbf{a}}^{1},\mathbf{a}^{1})$ we divide by $\sqrt{SSD_{2}}$, and therefore we only need to study the $SSD_{12}$ term in the prediction correlation and compare it to the $SSD_{y^{2}a^{1}}$ term in the accuracy. Since $y_{i}^{1}=a_{i}^{1}+e_{i}^{2}$ we see that $SSD_{12}=SSD_{y^{2}a^{1}}+SSD_{y^{2}e^{1}}$, and we obtain that

$$\rho(\widehat{\mathbf{a}}^{1},\mathbf{y}^{1})=c\rho(\widehat{\mathbf{a}}^{1},\mathbf{a}^{1})+cSSD_{y^{2}e^{1}}/\sqrt{SSD_{a^{1}}SSD_{2}},$$

where the proportionality constant $c=\sqrt{SSD_{a^{1}}/SSD_{1}}$ does not depend on the model for predicting the breeding values.

What remains to investigate is whether the term $SSD_{y^{2}e^{1}}/\sqrt{SSD_{a^{1}}SSD_{2}}$ is negligible or not. Since the effect are independent for different individuals, we obtain from the Law of Large Numbers that $SSD_{y^{2}e^{1}}/(n-1)\approx\sigma_{e,12}$, $SSD_{a^{1}}/(n-1)\approx\sigma_{a,11}$ and $SSD_{2}/(n-1)\approx\sigma_{a,22}+\sigma_{e,22}$, where we have used that genetic and residual effects are independent. Since $\sigma_{e,12}/\sqrt{\sigma_{a,11}(\sigma_{a,22}+\sigma_{e,22})}\neq0$, the term $SSD_{y^{2}e^{1}}/\sqrt{SSD_{a^{1}}SSD_{2}}$ is not negligible. Hence, we see that the predictive correlation is not approximately proportional to the accuracy of predicted breeding values. Therefore, the correlation between predicted breeding values and corrected phenotypes in this case would be not be a measure of accuracy of predicted breeding values.

Mrode R. Linear models for the prediction of animal breeding values. 3^rd^ edition. Wallingford, UK: CABI; 2013
