# Supplementary material for: Metabolomic-genomic prediction can improve prediction accuracy of breeding values for malting quality traits in barley
Source: Genet Sel Evol. 2023 Sep 5;55:61. doi: 10.1186/s12711-023-00835-w (PMC10478459; doi:10.1186/s12711-023-00835-w)
Supplement: Supplementary file 2 — Additional file 2: Table S1. Variance components from GBLUP and MBLUP1 for malting quality traits. Table S2. Relative variance components from GBLUP, MGBLUP1 and MGBLUP for malting quality traits. Table S3. Regression coefficients of predicted breeding values from whole data on predicted breeding values from partial data, for malting quality traits using GBLUP and MGBLUP (LOLO scheme). Table S4. Regression coefficients of predicted breeding values from whole data on predicted breeding values from partial data, for malting quality traits using GBLUP and MGBLUP (LOYO scheme). [file 12711_2023_835_MOESM2_ESM.docx]

**Additional file 2**

**Metabolomic-Genomic prediction can improve prediction accuracy of breeding values for malting quality traits in barley**

Xiangyu Guo^1, 2^, ﻿Pernille Sarup^3^, Ahmed Jahoor^3, 4^, Just Jensen^1^, Ole Fredslund Christensen^1*^

^1^ Center for Quantitative Genetics and Genomics, Aarhus University, 8000 Aarhus C, Denmark

^2^ Danish Pig Research Centre, Danish Agriculture & Food Council, 1609 Copenhagen V, Denmark

^3^ Nordic Seed A/S, 8300 Odder, Denmark

^4^ Department of Plant Breeding, The Swedish University of Agricultural Sciences, 2353 Alnarp, Sweden

^*^ Corresponding author:

**Tables**

**Table S1.** Variance components from GBLUP and MBLUP_1_ for malting quality traits

| Trait | Effect | GBLUP | MGBLUP_1_ |
| --- | --- | --- | --- |
| FS | ***m*** |  | 0.345 (0.079) |
|  | ***g*** | 0.014 (0.005) | 0.005 (0.003) |
|  | ***l*** | 0.005 (0.005) | 0.006 (0.004) |
|  | ***i_g_*** | 0.000 (0.004) | 0.000 (0.003) |
|  | ***i_l_*** | 0.026 (0.007) | 0.005 (0.005) |
|  | ***t*** | 0.058 (0.011) | 0.038 (0.008) |
|  | ***e*** | 0.132 (0.005) | 0.117 (0.005) |
|  | ***total*** | 0.236 (0.013) | 0.516 (0.077) |
| EY | ***m*** |  | 1.65 (0.49) |
|  | ***g*** | 0.29 (0.07) | 0.22 (0.05) |
|  | ***l*** | 0.00 (0.04) | 0.00 (0.04) |
|  | ***i_g_*** | 0.03 (0.03) | 0.02 (0.03) |
|  | ***i_l_*** | 0.22 (0.05) | 0.19 (0.05) |
|  | ***t*** | 0.17 (0.04) | 0.17 (0.05) |
|  | ***e*** | 0.97 (0.04) | 0.92 (0.04) |
|  | ***total*** | 1.68 (0.08) | 3.17 (0.48) |
| WC | ***m*** |  | 3.15 (0.38) |
|  | ***g*** | 0.14 (0.03) | 0.04 (0.01) |
|  | ***l*** | 0.06 (0.01) | 0.02 (0.00) |
|  | ***i_g_*** | 0.03 (0.01) | 0.01 (0.00) |
|  | ***i_l_*** | 0.03 (0.01) | 0.00 (0.00) |
|  | ***t*** | 0.07 (0.01) | 0.05 (0.01) |
|  | ***e*** | 0.16 (0.01) | 0.05 (0.007) |
|  | ***total*** | 0.50 (0.0300) | 3.32 (0.38) |
| BG | ***m*** |  | 111868 (13196) |
|  | ***g*** | 2625 (652) | 377 (204) |
|  | ***l*** | 1898 (337) | 901 (184) |
|  | ***i_g_*** | 1076 (346) | 569 (197) |
|  | ***i_l_*** | 882 (277) | 426 (182) |
|  | ***t*** | 975 (226) | 698 (180) |
|  | ***e*** | 4180 (162) | 1264 (232) |
|  | ***total*** | 11,636 (647) | 116,102 (12966) |
| WV | ***m*** |  | 0.0156 (0.0021) |
|  | ***g*** | 0.0004 (0.0001) | 0.0001 (0.0000) |
|  | ***l*** | 0.0003 (0.0001) | 0.0002 (0.0000) |
|  | ***i_g_*** | 0.0002 (0.0001) | 0.0001 (0.0000) |
|  | ***i_l_*** | 0.0001 (0.0000) | 0.0000 (0.0000) |
|  | ***t*** | 0.0008 (0.0002) | 0.0008 (0.0002) |
|  | ***e*** | 0.0008 (0.0000) | 0.0003 (0.0000) |
|  | ***total*** | 0.0025 (0.0002) | 0.0171 (0.0020) |

Trait: FS = filtering speed, EY = extract yield, WC = wort color, BG = beta-glucan, WV = wort viscosity;

Method: GBLUP = genomic best linear unbiased prediction, MGBLUP_1_ = metabolomics genomic best linear unbiased prediction, step 1.

Effects: ***g*** is genomic effects, ***l*** is line effect, ***i_g_*** is genotype by environmental effects, ***i_l_*** is line by environmental effects, ***t*** is malt-mash effects, and ***e*** is residuals. Columns show estimated variance components with numbers in brackets being the standard errors on the estimates.

**Table S2.** Relative variance components from GBLUP, MGBLUP_1_ and MGBLUP for malting quality traits.

| Trait | Effect | GBLUP | MGBLUP_1_ | MGBLUP |
| --- | --- | --- | --- | --- |
| FS | ***m*** |  | 0.668 (0.055) |  |
|  | ***g*** | 0.060 (0.021) | 0.010 (0.005) | 0.123 (0.008) |
|  | ***l*** | 0.021 (0.022) | 0.010 (0.007) | 0.052 (0.009) |
|  | ***i_g_*** | 0.000 (0.017) | 0.000 (0.006) | 0.105 (0.008) |
|  | ***i_l_*** | 0.111 (0.030) | 0.001 (0.010) | 0.135 (0.012) |
|  | ***t*** | 0.243 (0.037) | 0.073 (0.019) | 0.187 (0.020) |
|  | ***e*** | 0.561 (0.033) | 0.227 (0.038) | 0.398 (0.039) |
| EY | ***m*** |  | 0.522 (0.078) |  |
|  | ***g*** | 0.175 (0.035) | 0.069 (0.020) | 0.187 (0.023) |
|  | ***l*** | 0.000 (0.023) | 0.000 (0.011) | 0.049 (0.013) |
|  | ***i_g_*** | 0.015 (0.019) | 0.007 (0.009) | 0.028 (0.011) |
|  | ***i_l_*** | 0.133 (0.033) | 0.059 (0.019) | 0.093 (0.021) |
|  | ***t*** | 0.099 (0.023) | 0.054 (0.016) | 0.118 (0.018) |
|  | ***e*** | 0.578 (0.030) | 0.289 (0.048) | 0.525 (0.050) |
| WC |  |  | 0.948 (0.009) |  |
|  | ***g*** | 0.279 (0.047) | 0.013 (0.003) | 0.283 (0.004) |
|  | ***l*** | 0.125 (0.026) | 0.005 (0.002) | 0.123 (0.002) |
|  | ***i_g_*** | 0.058 (0.02) | 0.004 (0.002) | 0.044 (0.002) |
|  | ***i_l_*** | 0.061 (0.021) | 0.000 (0.001) | 0.087 (0.002) |
|  | ***t*** | 0.146 (0.026) | 0.014 (0.003) | 0.095 (0.003) |
|  | ***e*** | 0.331 (0.022) | 0.016 (0.004) | 0.367 (0.004) |
| BG | ***m*** |  | 0.963 (0.0065) |  |
|  | ***g*** | 0.226 (0.047) | 0.003 (0.002) | 0.267 (0.002) |
|  | ***l*** | 0.163 (0.030) | 0.008 (0.002) | 0.095 (0.002) |
|  | ***i_g_*** | 0.092 (0.029) | 0.005 (0.002) | 0.047 (0.002) |
|  | ***i_l_*** | 0.076 (0.024) | 0.004 (0.002) | 0.039 (0.002) |
|  | ***t*** | 0.084 (0.018) | 0.006 (0.002) | 0.1230 (0.002) |
|  | ***e*** | 0.359 (0.023) | 0.011 (0.003) | 0.428 (0.003) |
| WV | ***m*** |  | 0.912 (0.015) |  |
|  | ***g*** | 0.152 (0.038) | 0.005 (0.003) | 0.235 (0.003) |
|  | ***l*** | 0.121 (0.023) | 0.010 (0.002) | 0.088 (0.003) |
|  | ***i_g_*** | 0.072 (0.022) | 0.008 (0.002) | 0.034 (0.003) |
|  | ***i_l_*** | 0.023 (0.017) | 0.001 (0.002) | 0.035 (0.002) |
|  | ***t*** | 0.314 (0.045) | 0.044 (0.010) | 0.145 (0.010) |
|  | ***e*** | 0.317 (0.025) | 0.020 (0.004) | 0.464 (0.0049) |

Trait: FS = filtering speed, EY = extract yield, WC = wort color, BG = beta-glucan, WV = wort viscosity;

Method: GBLUP = genomic best linear unbiased prediction, MGBLUP1 = metabolomics genomic best linear unbiased prediction, step 1.

Effects: ***g*** is genomic effects, ***l*** is line effect, ***i_g_*** is genotype by environmental effects, ***i_l_*** is line by environmental effects, ***t*** is malt-mash effects, and ***e*** is residuals. Columns show estimates of relative variance components (RVCs), which are the proportions of variances for each traits, where those for MGBLUP are computed using the formula ${RVC=c}_{m}^{2}RVC_{1}+RVC_{2}$, with $RVC_{1}$ and $RVC_{2}$, denoting the proportions of total variance in the step 1 and 2 for MGBLUP, respectively. Standard errors on RVCs are shown in brackets.

## Table S3. Regression coefficients of predicted breeding values from whole data on predicted breeding values from partial data, for malting quality traits using GBLUP and MGBLUP (LOLO scheme).

| Trait | GBLUP-g/gp | MGBLUP-g/gm | MGBLUP-gm/gmp | MGBLUP-g/gmp |
| --- | --- | --- | --- | --- |
| FS | 0.99 | 0.99 | 0.99 | 0.99 |
| EY | 1.00 | 1.01 | 1.00 | 1.01 |
| WC | 1.00 | 1.00 | 1.01 | 0.99 |
| BG | 0.99 | 0.99 | 1.02 | 1.00 |
| WV | 0.99 | 0.99 | 1.02 | 1.00 |

GBLUP = genomic best linear unbiased prediction. MGBLUP = metabolomic-genomic best linear unbiased prediction. LOLO = leave one line out. Trait: FS = filtering speed, EY = extract yield, WC = wort color, BG = beta-glucan, WV = wort viscosity. GBLUP-g/gp is the regression coefficient of predicted breeding values on focal individuals from GBLUPgp on predicted breeding values from GBLUPp, where GBLUPg is GBLUP incorporating genotypes on focal individuals, and GBLUPgp is GBLUP incorporating genotypes and phenotypes on focal individuals. MGBLUP-g/gm, MGBLUP-gm/gmp and MGBLUP-g/gmp are similarly defined with “gm” denoting genotypes and metabolomics on focal individuals, and “gmp” denoting genotypes, metabolomics and phenotypes on focal individuals.

## Table S4. Regression coefficients of predicted breeding values form whole data on predicted breeding values from partial data, for malting quality traits using GBLUP and MGBLUP (LOYO scheme).

| Trait | GBLUP-g/gp | MGBLUP-g/gm | MGBLUP-gm/gmp | MGBLUP-g/gmp |
| --- | --- | --- | --- | --- |
| FS | 0.98 | 1.16 | 1.02 | 1.19 |
| EY | 0.96 | 1.01 | 1.00 | 0.99 |
| WC | 0.85 | 0.89 | 1.04 | 0.84 |
| BG | 0.83 | 1.06 | 0.96 | 1.01 |
| WV | 0.73 | 1.06 | 0.87 | 0.86 |

GBLUP = genomic best linear unbiased prediction. MGBLUP = metabolomic-genomic best linear unbiased prediction. LOLO = leave one line out. Trait: FS = filtering speed, EY = extract yield, WC = wort color, BG = beta-glucan, WV = wort viscosity. GBLUP-g/gp is the regression coefficient of predicted breeding values on focal individuals from GBLUPgp on predicted breeding values from GBLUPp, where GBLUPg is GBLUP incorporating genotypes on focal individuals, and GBLUPgp is GBLUP incorporating genotypes and phenotypes on focal individuals. MGBLUP-g/gm, MGBLUP-gm/gmp and MGBLUP-g/gmp are similarly defined with “gm” denoting genotypes and metabolomics on focal individuals, and “gmp” denoting genotypes, metabolomics and phenotypes on focal individuals.
