# Supplementary material for: Metabolomic-genomic prediction can improve prediction accuracy of breeding values for malting quality traits in barley
Source: Genet Sel Evol. 2023 Sep 5;55:61. doi: 10.1186/s12711-023-00835-w (PMC10478459; doi:10.1186/s12711-023-00835-w)
Supplement: Supplementary file 3 — Additional file 3: Figure S1. Proportion of total phenotypic variance explained by each component in malting quality traits. [file 12711_2023_835_MOESM3_ESM.docx]

**Additional file 3**

**Metabolomic-Genomic prediction can improve prediction accuracy of breeding values for malting quality traits in barley**

Xiangyu Guo^1, 2^, ﻿Pernille Sarup^3^, Ahmed Jahoor^3, 4^, Just Jensen^1^, Ole Fredslund Christensen^1*^

^1^ Center for Quantitative Genetics and Genomics, Aarhus University, 8000 Aarhus C, Denmark

^2^ Danish Pig Research Centre, Danish Agriculture & Food Council, 1609 Copenhagen V, Denmark

^3^ Nordic Seed A/S, 8300 Odder, Denmark

^4^ Department of Plant Breeding, The Swedish University of Agricultural Sciences, 2353 Alnarp, Sweden

^*^ Corresponding author:

**Figures**


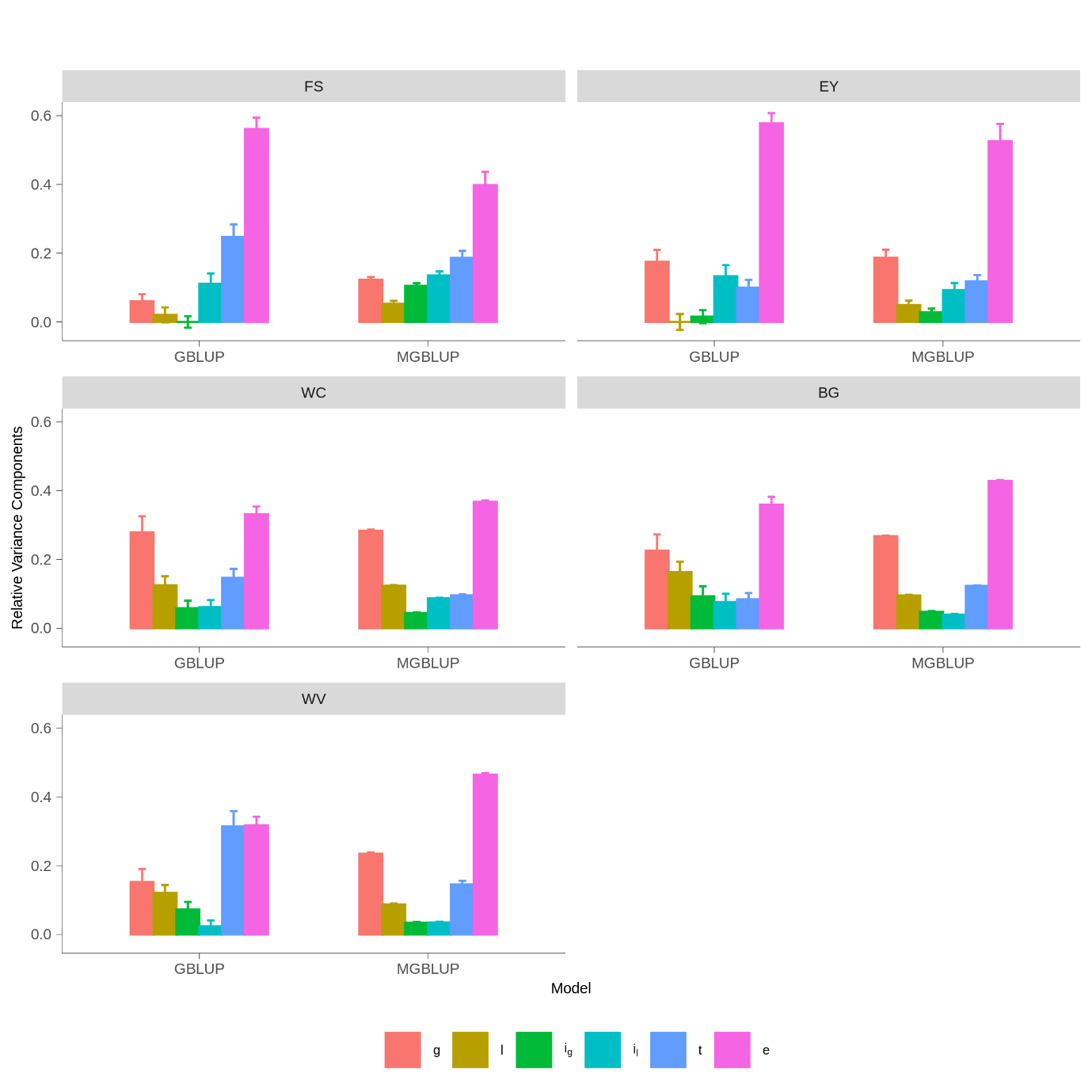


## Figure S1 Proportion of total phenotypic variance explained by each component in malting quality traits

Trait: FS = filtering speed, EY = extract yield, WC = wort color, BG = beta-glucan, WV = wort viscosity; y-axis is relative variance component; g is relative variance of genomic effects, l is relative variance of line effect, i_g_ is relative variance of genotype by environmental effects, i_l_ is relative variance of line by environmental effects, ***t*** is relative variance of malt-mash effects, and e is relative variance of residuals. Method: GBLUP = genomic best linear unbiased prediction, MGBLUP = metabolomic-genomic best linear unbiased prediction.
