# Supplementary material for: Metabolomic-genomic prediction can improve prediction accuracy of breeding values for malting quality traits in barley
Source: Genet Sel Evol. 2023 Sep 5;55:61. doi: 10.1186/s12711-023-00835-w (PMC10478459; doi:10.1186/s12711-023-00835-w)
Supplement: Supplementary file 4 — Additional file 4: Text S2. Deviation between empirical variance and total phenotypic variance. Derivations show that the variance of empirical variance is much larger for MGBLUP than for GBLUP. [file 12711_2023_835_MOESM4_ESM.docx]

**Additional file 4**

**Metabolomic-Genomic prediction can improve prediction accuracy of breeding values for malting quality traits in barley**

Xiangyu Guo^1, 2^, ﻿Pernille Sarup^3^, Ahmed Jahoor^3, 4^, Just Jensen^1^, Ole Fredslund Christensen^1*^

^1^ Center for Quantitative Genetics and Genomics, Aarhus University, 8000 Aarhus C, Denmark

^2^ Danish Pig Research Centre, Danish Agriculture & Food Council, 1609 Copenhagen V, Denmark

^3^ Nordic Seed A/S, 8300 Odder, Denmark

^4^ Department of Plant Breeding, The Swedish University of Agricultural Sciences, 2353 Alnarp, Sweden

^*^ Corresponding author:

**Deviation between empirical variance and total phenotypic variance**

In the main paper, for several traits, we saw a result about the total phenotypic variance being much larger than the empirical variance (in the main paper termed “variance of phenotype”), which was unexpected. Here, we aim to provide some explanations on the relationship between these two terms.

First, we derive total phenotypic variance as an expectation of empirical variance, $S_{y}^{2}$. Second, we show a formula for the variance of the empirical variance, $Var(S_{y}^{2})$. Third, we show that $Var(S_{y}^{2})$ is much larger for the metabolomics-genomic model, than for the genomic model, which then implies that the deviation between the empirical variance and the total phenotypic variance for the metabolomics-genomic model is allowed to be much larger than the deviation between the empirical variance and the total phenotypic variance for the genomic model.

**Total phenotypic variance**

Here, we show a formula that expresses the expected value of the empirical variance as a function of the parameters in a model. This expression is the total phenotypic variance in the model.

We consider a simple model with a constant mean

$$\mathbf{Y}=\mu\mathbf{1}+\mathbf{v}+\mathbf{e},$$

where $var(\mathbf{v})=\sigma_{q}^{2}\mathbf{Q}$, $var(\mathbf{e})=\sigma_{e}^{2}\mathbf{I}$, and $\mathbf{\Sigma}=var(\mathbf{y})=var(\mathbf{v})+var(\mathbf{e})=\sigma_{q}^{2}\mathbf{Q}+\sigma_{e}^{2}\mathbf{I}$.

The empirical variance

$$S_{y}^{2}=\sum_{i} (y_{i}-\bar{y})^{2}/(n-1)=\mathbf{y}^{T}\mathbf{Py}/(n-1),$$

where $\mathbf{P}=\mathbf{I}-\mathbf{11}^{T}/n$, i.e. the projection matrix into the orthogonal space of the mean space. The expectation of $S_{y}^{2}$ can therefore be computed using a formula for the expectation of a quadratic form, $E[\mathbf{y}^{T}\mathbf{Py}]=\text{Tr}(\mathbf{P\Sigma})+(\mu\mathbf{1})^{T}\mathbf{P}(\mu\mathbf{1}))$, where $\text{Tr}$ is the trace of the matrix. From this expression, since $\mathbf{1}^{T}\mathbf{P1}=0$ and $\text{tr}(\mathbf{P})=n-1$, we obtain that

$$E[S_{y}^{2}]=\sigma_{q}^{2}Tr(\mathbf{PQ})/(n-1)+\sigma_{e}^{2}=\sigma_{q}^{2}(\overline{\delta Q}-\overline{oQ})+\sigma_{e}^{2},$$

where $\overline{\delta Q}$ is the average of diagonal elements, $\overline{oQ}$ is the average of off-diagonal elements, and we have used that $\text{Tr}(\mathbf{Q})=n\overline{\delta Q}$ and $\text{Tr}((\mathbf{11}^{T}/n)\mathbf{Q})=\overline{\delta Q}+(n-1)\overline{oQ}$. This expression for the expectation of the empirical variance is the total phenotypic variance.

**Remark 1:** For type of similarity/relationship matrix as in VanRaden (2008), $\mathbf{Q}=\sum_{j} (\mathbf{m}_{j}-c_{j}\mathbf{1})(\mathbf{m}_{j}-c_{j}\mathbf{1})^{T}/s$ where $c_{j}$’s are centering terms and $s$ is a scaling constant. In case $c_{j}=\bar{m}_{j}$ is used, then $\overline{Q}=0$, and hence $\overline{oQ}=\overline{-\delta Q}/(n-1)$, and the formula simplifies.

**Formula for variance of** $S_{y}^{2}$

In the derivation below, we will use the formula $Cov(y^{2},z^{2})=2Cov(y,z)^{2}$ which holds for $(y,z)$ being multivariate normal distributed. For simplicity of notation we will denote $\mathbf{V}=\sigma_{q}^{2}\mathbf{Q}+\sigma_{e}^{2}\mathbf{I}$ for the variance-covariance matrix of $y$.

First, we rewrite $S_{y}^{2}$ as

$$S_{y}^{2}=\sum_{i} y_{i}^{2}/(n-1)-\bar{y}^{2}n/(n-1).$$

From this we obtain

$$Var(S_{y}^{2})=\sum_{i,j} Cov(y_{i}^{2},y_{j}^{2})/(n-1)^{2}-2\sum_{i} Cov(y_{i}^{2},\bar{y}^{2})n/(n-1)^{2}+Var(\bar{y}^{2})(n/(n-1))^{2}.$$

Using the $Cov(y^{2},z^{2})=2Cov(y,z)^{2}$ formula we obtain

$$Var(S_{y}^{2})=2\sum_{i,j} (V_{ij})^{2}/(n-1)^{2}-4\sum_{i} (\bar{V}_{i})^{2}n/(n-1)^{2}+2\bar{V}^{2}n^{2}/(n-1)^{2}$$

where $\bar{V}_{i}$ the mean of the $i$’th row of $\mathbf{V}$ (due to symmetry of $\mathbf{V}$ also equal to the mean of the $i$’th column) , and $\bar{V}$ is the mean of all elements of $\mathbf{V}$. This equals

$$Var(S_{y}^{2})=2\sum_{i,j} (V_{ij}-\bar{V}_{i})^{2}/(n-1)^{2}-2\sum_{i} (\bar{V}_{i}-\bar{V})^{2}n/(n-1)^{2}.$$

The sums of squares in this expression equal

$$\sum_{i,j} (V_{ij}-\bar{V}_{i})^{2}=(\sigma_{q}^{2})^{2}\sum_{i,j} (Q_{ij}-\bar{Q}_{i})^{2}+2\sigma_{q}^{2}\sigma_{e}^{2}(\overline{dQ}-\bar{Q})n+(\sigma_{e}^{2})^{2}(n-1),$$

$$\sum_{i} (\bar{V}_{i}-\bar{V})^{2}=(\sigma_{q}^{2})^{2}\sum_{i} (\bar{Q}_{i}-\bar{Q})^{2}+0+0.$$

For a vanRaden type of similarity matrix where $\mathbf{Q}=\mathbf{MM}^{T}/s$ with column means of $\mathbf{M}$ being zero then $\bar{Q}_{i}=\bar{Q}=0$, and using this, the above formula gives

$$Var(S_{y}^{2})=2(\sigma_{q}^{2})^{2}\overline{Q^{2}}n^{2}/(n-1)^{2}+4\sigma_{q}^{2}\sigma_{e}^{2}\overline{\partial Q}n/(n-1)^{2}+2(\sigma_{e}^{2})^{2}/(n-1).$$

where $\overline{Q^{2}}=\sum_{i,j} (Q_{ij})^{2}/n^{2}$. When $n$ is large, $\bar{Q}$ is strictly positive, and the magnitude of $\sigma_{q}^{2}$ is similar to or larger than $\sigma_{e}^{2}$, then

$$Var(S_{y}^{2})\approx2(\sigma_{q}^{2})^{2}\overline{Q^{2}}.$$

**Parameters for the metabolomics model compared to genomic model**

Here, we show that $Var(S_{y}^{2})$ is much larger when $\mathbf{Q}$ is the metabolomics similarity matrix compared to $\mathbf{Q}=\mathbf{G}$ being the genomic relationship matrix. First, as shown in results in the main section, $\sigma_{q}^{2}$ is larger for the metabolomics similarity matrix compared to the genomic relationship matrix. Second, $\overline{Q^{2}}=0.45$for the metabolic similarity matrix is six times larger than $\overline{G^{2}}=0.07$. Together this shows that $Var(S_{y}^{2})$ is much larger for MGBLUP1 than for GBLUP, which implies that a larger deviation of total phenotypic variance from empirical variance is possible for MGBLUP1 compared to GBLUP.

VanRaden PM (2008). Efficient methods to compute genomic predictions. *J Dairy Sci* **91**: 4414-4423.
